# Weight discrimination and cardiometabolic health in underrepresented US adults

**DOI:** 10.1007/s10865-026-00696-w

**Published:** 2026-07-01

**Authors:** Mary A. Gerend, Anna W. Lu

**Affiliations:** Department of Behavioral Sciences and Social Medicine, College of Medicine, Florida State University, 1115 West Call Street, Tallahassee, FL 32306-4300 USA

**Keywords:** Cardiovascular diseases, Metabolic diseases, Cardiometabolic risk factors, Weight prejudice, Obesity, Social determinants of health

## Abstract

This study investigated the association between perceived weight discrimination and cardiometabolic health. A demographically diverse sample of 2632 US adults (36% Black/African American; 36% Hispanic/Latino; 29% sexual minority) completed a cross-sectional online survey. Linear regression was used to assess the association between perceived weight discrimination (assessed with the Stigmatizing Situations Survey-Brief) and cardiometabolic risk (indicated by the number of cardiometabolic conditions reported: high blood pressure, high cholesterol, heart disease, stroke, kidney disease, or diabetes), adjusting for body mass index and demographic characteristics. Cigarette smoking, alcohol use, and sleep disturbance were investigated as mediators. Perceived weight discrimination was associated with higher cardiometabolic risk. Further, greater exposure to weight discrimination was associated with higher likelihood of smoking, problematic drinking behavior, and disrupted sleep, and those behaviors in turn predicted higher cardiometabolic risk. Findings underscore the importance of addressing the negative health consequences of weight discrimination in cardiometabolic disease prevention programs.

## Introduction

Weight-related discrimination is a key social determinant of health that may increase risk of cardiometabolic disease (Pearl et al., 2024; Puhl & Heuer, 2010). Weight discrimination refers to negative or unfair treatment that is experienced by individuals because of their body weight or size. Although individuals of any weight or size can experience weight discrimination, people with high body weight or large body size are most likely to be targeted because of their devalued status in society (Spahlholz et al., 2016). Encounters with weight-related discrimination can range from subtle instances of differential treatment (e.g., verbal and nonverbal insults or slights known as “microaggressions”) to more overt actions (e.g., being denied employment or a promotion). Weight discrimination has been documented in a variety of settings including education and employment, personal relationships, and health care (Pearl, 2018; Puhl & Heuer, 2009, 2010; Spahlholz et al., 2016). A meta-analysis found that nearly 20% of individuals with class I obesity (body mass index [BMI] = 30–34.9 kg/m^2^) and over 40% of individuals with class II obesity or higher (BMI ≥ 35 kg/m^2^) report having experienced weight discrimination in their lifetime (Spahlholz et al., 2016).

Theoretical models of discrimination and health propose that, like other forms of discrimination (e.g., racism, sexism), exposure to weight discrimination activates a cascade of psychological (e.g., perceived stress, negative emotions), physiological (e.g., cardiovascular reactivity, cortisol secretion), and behavioral (e.g., maladaptive eating behavior, smoking) stress responses that lead to poor mental and physical health outcomes (Hatzenbuehler, 2009; Major et al., 2018; Pascoe & Richman, 2009; Pascoe et al., 2022; Tomiyama, 2014; Williams & Mohammed, 2009; Williams et al., 2019). Notably, a feedback loop can form such that people with high body weight are more likely to experience weight discrimination, which in turn can trigger stress responses (e.g., comfort eating, cortisol secretion) that contribute to further weight gain, central adiposity, and the maintenance of obesity (Tomiyama, 2014). Over time, these stress responses are believed to contribute to cardiometabolic dysfunction (e.g., hypertension, insulin resistance) and disease (e.g., heart disease, stroke, diabetes).

Consistent with the conceptual model described previously, associations between weight discrimination and indicators of poor cardiometabolic health have been documented. For instance, perceived weight discrimination is associated with longitudinal increases in weight gain and waist circumference (Jackson et al., 2014; Sutin & Terracciano, 2013). Likewise, using longitudinal data from the Midlife in the United States Study, Vadiveloo and Mattei found that perceived weight discrimination was associated with twice the risk of high allostatic load and worse lipid/metabolic regulation, glucose metabolism, and inflammation (Vadiveloo & Mattei, 2017). Cross-sectional studies have linked perceived weight discrimination to metabolic syndrome, a cluster of risk factors involving impaired glucose tolerance, decreased high-density lipoprotein cholesterol, high blood pressure, high triglycerides, and abdominal obesity, as well as several chronic conditions including arteriosclerosis, high cholesterol, diabetes, myocardial infarction, and minor heart conditions (e.g., angina pectoris, tachycardia) (Adil et al., 2022; Udo et al., 2016). Experimental research has shown that exposure to weight discrimination can increase blood pressure (Major et al., 2012) and salivary cortisol levels (Himmelstein et al., 2015; Schvey et al., 2014). Of note, these associations generally hold when models are adjusted for BMI, demonstrating that interpersonal experiences with weight discrimination confer independent risk to health beyond physiological effects associated with BMI. A key limitation of previous research, however, is that most of these studies are limited to samples with largely non-Hispanic White participants and thus findings may not generalize to individuals from underrepresented groups.

Mounting evidence also supports behavioral stress responses (i.e., health risk behaviors) as one important pathway through which exposure to weight discrimination may harm cardiometabolic health (Pascoe & Richman, 2009; Pascoe et al., 2022). Unhealthy eating behaviors and physical inactivity have received the most attention in the literature, as these behaviors are often triggered by exposure to weight discrimination (Pearl et al., 2021; Puhl et al., 2020; Vartanian & Porter, 2016). For example, numerous studies have documented a relationship between weight discrimination and maladaptive eating behavior (e.g., binge eating and restrictive eating behaviors) (Puhl et al., 2020; Sutin et al., 2016; Vartanian & Porter, 2016).

Despite their well-established connections to cardiometabolic disease (Chaput & Stranges, 2025; Direksunthorn, 2025; Gallucci et al., 2020; Messner & Bernhard, 2014; Meza et al., 2022; Piano et al., 2025), significantly less research has explored cigarette smoking, alcohol use, and disturbed sleep as possible mechanisms underlying the association between weight discrimination and poor cardiometabolic health. Smoking induces endothelial dysfunction, oxidative stress, inflammation, pro-thrombotic states, adverse lipid alterations, and insulin resistance, thus accelerating atherosclerosis and intensifying risk of cardiovascular disease and other adverse cardiometabolic outcomes (Gallucci et al., 2020; Messner & Bernhard, 2014). Alcohol consumption disrupts hepatic metabolism, which promotes oxidative stress, inflammation, dyslipidemia, and insulin resistance (Meza et al., 2022). Previous studies indicate that while low alcohol intake (e.g., no more than 1 or 2 drinks a day for women and men, respectively) shows mixed observational associations, higher consumption (e.g., ≥ 3 drinks a day) is consistently linked to increased cardiometabolic risk (Piano et al., 2025). Inadequate or disrupted sleep impairs glucose metabolism, decreases insulin sensitivity, elevates sympathetic nervous system activity, activates stress hormone release, and promotes systemic inflammation, increasing the risk for hypertension, type 2 diabetes, cardiovascular events, and all-cause mortality (Chaput & Stranges, 2025; Direksunthorn, 2025).

Previous research has linked perceived discrimination with smoking, alcohol consumption, and sleep-related problems (Lewis et al., 2014; Slopen et al., 2016). Although less research has investigated these behaviors in the context of weight-related discrimination, evidence for perceived weight discrimination’s connection with smoking (Gerend et al., 2023; Himmelstein et al., 2019; Sutin & Terracciano, 2017), alcohol use (Gerend et al., 2023; Klinck et al., 2020; Puhl et al., 2019), and disturbed sleep (Gerend et al., 2023; Himmelstein et al., 2019; Lee et al., 2021) is accruing. To our knowledge, however, no studies have examined these behaviors as potential mediators of the association between weight discrimination and cardiometabolic health outcomes.

To address these gaps, the primary objectives of the present study were to (1) examine the association between perceived weight discrimination and cardiometabolic health in a demographically diverse sample of US adults and (2) investigate cigarette smoking, alcohol use, and sleep disturbance as behavioral mechanisms potentially underlying this association. A secondary objective was to assess whether the strength of the association between perceived weight discrimination and cardiometabolic health was moderated by key demographic characteristics (i.e., race, ethnicity, gender, and sexual orientation). To achieve these objectives, we recruited a large sample of US men and women with representation across the broad BMI spectrum. In addition, we used quota sampling to ensure inclusion of a disproportionately high proportion of respondents who identified as Black or African American, Hispanic or Latino, and/or a sexual minority. Thus, this study allows us to assess whether the negative cardiometabolic health consequences of weight discrimination are observed in a diverse sample of participants and whether effects are stronger in some demographic groups relative to others. We hypothesized that more frequent exposure to weight discrimination would be associated with worse cardiometabolic health, and that this association would be mediated by smoking, alcohol use, and sleep disturbance. Given limited findings on whether the strength of the association between weight discrimination and cardiometabolic health outcomes differs by demographic characteristics, all moderation analyses were treated as exploratory.

## Methods

### Participants and procedure

Data were drawn from a large cross-sectional study on weight stigma and health that was conducted in 2021 using Dynata’s online sampling platform. A detailed description of the procedure is provided elsewhere (Gerend et al., 2023). The purpose of the overarching study was to assess associations between weight stigma and health-related outcomes in demographic subgroups who have historically been less well represented in weight stigma research, both to broaden the literature and generate hypotheses for future research. Variables analyzed in the current study included perceived weight discrimination, cigarette smoking, alcohol use, sleep disturbance, and cardiometabolic health. Eligibility was limited to US adults aged 18–64 years who identified as a cisgender man or woman (i.e., gender identity aligned with sex assigned at birth) and had a BMI between 12 and 70 kg/m^2^. Because previous studies on weight stigma have primarily included White participants without considering sexual orientation, we purposely oversampled participants from minoritized groups. Quota sampling was used to ensure that at least a third of the sample identified as Black or African American, a third identified as Hispanic or Latino, and a quarter identified as a sexual minority (e.g., gay, lesbian, bisexual). Our target sample size was 2500 participants, which provided sufficient statistical power for the proposed analyses, including mediation analyses (Fritz & Mackinnon, 2007). This study was performed in line with the principles of the Declaration of Helsinki and was approved by the university Institutional Review Board. All participants provided informed consent before completing the online survey.

### Measures

Perceived weight discrimination was assessed with the Stigmatizing Situations Survey-Brief (SSI-B) (Vartanian, 2015), a 10-item version of the original 50-item scale (Myers & Rosen, 1999). Participants indicated how frequently in their lifetime they experienced various “situations that people encounter because of their weight” (e.g., “Having a doctor recommend a diet even if you did not come in to discuss weight loss.” “Not being hired because of your weight, shape or size.”). Following Puhl and Brownell (Puhl & Brownell, 2006), items were rated on a 4-point scale (0 = *never*; 1 = *once*; 2 = *more than once*; 3 = *multiple times*). The ten items were averaged to create a total score, with higher scores indicating more frequent exposure to weight discrimination (α = .92).

Cardiometabolic health was assessed using items from the Centers for Disease Control and Prevention’s Behavioral Risk Factor Surveillance System (BRFSS) (Centers for Disease Control & Prevention, 2020): “Has a doctor, nurse, or other health professional ever told you that you had: (1) high blood pressure, (2) high cholesterol, (3) a heart condition (e.g., heart attack, angina, coronary heart disease), (4) kidney disease (not including kidney stones, bladder infection or incontinence), (5) stroke, or (6) diabetes.” Response options included “no” (0), “yes” (1), or “don’t know.” None of the participants selected “don’t know.” Items were summed to create an index that ranged from 0 to 6, with higher scores indicating greater cardiometabolic risk.

Cigarette smoking was assessed with two items from the BRFSS (Centers for Disease Control & Prevention, 2020): “Have you smoked at least 100 cigarettes in your entire life?” and “Do you now smoke cigarettes every day, some days, or not at all?” Following coding guidelines from the BRFSS, responses to these two questions were used to categorize participants as 0 = *never smokers*, 1 = *former smokers*, or 2 = *current smokers*. Participants who had not smoked at least 100 cigarettes in their lifetime were coded as never smokers. Participants who had smoked at least 100 cigarettes in their lifetime but were not currently smoking were coded as former smokers. Participants who had smoked at least 100 cigarettes in their lifetime and currently smoked some days or every day were coded as current smokers.

Alcohol use was assessed with three alcohol consumption questions from the Alcohol Use Disorders Identification Test (AUDIT-C), a screening test designed to identify individuals who engage in heavy drinking and/or show signs of active alcohol abuse or dependence (Bush et al., 1998). Participants answered three questions and each response option was assigned points following scoring instructions: “How often did you have a drink containing alcohol in the past year? Consider a drink to be a can or bottle of beer, a glass of wine, a wine cooler, or one cocktail or a shot of hard liquor (like scotch, gin, vodka).” Response options were never (0 points); monthly or less (1 point); 2 to 4 times a month (2 points); 2 to 3 times a week (3 points); 4 to 5 times a week (4 points); or six or more times a week (4 points). “How many drinks did you have on a typical day when you were drinking in the past year?” Response options were 0 drinks (0 points); 1 to 2 drinks (0 points); 3 to 4 drinks (1 point); 5 to 6 drinks (2 points); 7 to 9 drinks (3 points); or 10 or more drinks (4 points). “How often did you have 6 or more drinks on one occasion in the past year?” Response options were never (0 points); less than monthly (1 point); monthly (2 points); weekly (3 points); or daily or almost daily (4 points). Scores were summed to create a total score that ranged from 0 to 12, with higher scores indicating more problematic alcohol use (α = .82). The survey also included the 7-item Patient-Reported Outcomes Measurement Information System (PROMIS) alcohol measure, which assesses problematic drinking in the past 30 days (e.g., “I had trouble controlling my drinking”) (Pilkonis et al., 2016). Because the two alcohol use measures were highly correlated (r = .70) and resulted in nearly identical findings, only results using the AUDIT-C are included in this article.

Sleep disturbance was assessed with the 6-item PROMIS short form (Yu et al., 2012), which was designed to assess severity of *current* sleep-related problems. Participants rated their sleep quality (1 = *very poor* to 5 = *very good*) and the extent to which they experienced various sleep-related problems (e.g., difficulty falling asleep; restless sleep) in the past seven days using a 5-point scale (1 = *not at all* to 5 = *very much*). After reverse scoring relevant items, the six items were averaged to create a score, with higher scores indicating more sleep disturbance (α = .89).

Participants also reported demographic characteristics (i.e., age, sex, gender, race, ethnicity, sexual orientation, education, and income) and current height and weight. Height and weight were used to compute BMI (kg/m^2^).

### Statistical analysis

The independent variable of interest was perceived weight discrimination as indicated by SSI-B score. The primary outcome variable was the cardiometabolic risk index, indicated by the number of cardiometabolic conditions reported by each participant. Secondary outcomes were the six cardiometabolic conditions (i.e., high blood pressure, high cholesterol, a heart condition, kidney disease, stroke, and diabetes) considered individually.

Descriptive statistics were computed for sample characteristics. Descriptive statistics and correlations among perceived weight discrimination, proposed mediators (i.e., smoking history, alcohol use, and sleep disturbance), diagnosis with each of the six conditions, and the cardiometabolic risk index were also estimated. We used multivariable linear regression with listwise deletion to predict the primary outcome from perceived weight discrimination, adjusting for race (Black = 1; non-Black = 0), ethnicity (Latino = 1; non-Latino = 0), gender (woman = 1; man = 0), sexual orientation (sexual minority = 1; straight/heterosexual = 0), age, education, income, and BMI. We used multivariable logistic regression to predict each secondary outcome from perceived weight discrimination while adjusting for all covariates. To test whether smoking history, alcohol use, and sleep disturbance mediated the association between perceived weight discrimination and cardiometabolic risk, we conducted a parallel mediation analysis using the PROCESS macro in SPSS (Hayes, 2022), again controlling for BMI and demographic characteristics. We specified a linear regression model and estimated indirect effects using percentile bootstrap confidence intervals based on 5,000 samples. To assess whether the association between perceived weight discrimination and cardiometabolic risk was moderated by race, ethnicity, gender, or sexual orientation, we conducted an exploratory multivariable linear regression analysis in which cardiometabolic risk was predicted from perceived weight discrimination, the four demographic variables of interest (i.e., race: Black or non-Black; ethnicity: Latino or non-Latino; gender: woman or man; and sexual orientation: sexual minority or straight/heterosexual), interactions between weight discrimination and each of these four demographic variables, and the remaining covariates (i.e., age, education, income, and BMI). Variables were centered prior to analysis. Significant interactions were followed with simple effects tests to assess the pattern of the interaction.

## Results

Sample characteristics for the 2,632 participants who completed the survey are presented in Table 1. Participant age ranged from 18 to 64 years with a mean of 36.9 years (SD = 12.5). Approximately 50% of the sample were women/female sex assigned at birth. With respect to race and ethnicity, 36% of the sample identified as Black or African American and 36% of the sample identified as Latino or Hispanic. Nearly 30% of participants identified as a sexual minority. The median annual household income fell between $35,000–$49,999. Approximately 30% of the sample had a high school diploma or equivalent and 26% attended some college. The mean BMI for the sample was 27.9 kg/m^2^ (SD = 8.2), with 27% and 32% meeting BMI criteria for overweight and obesity, respectively.

**Table 1:** Sample characteristics (N = 2632)

	Mean (SD) or N (%)
Age (years)	36.9 (12.5)
Gender	
Man	1327 (50)
Woman	1305 (50)
Sex	
Male	1327 (50)
Female	1305 (50)
Latino or Hispanic ethnicity	
No	1690 (64)
Yes	942 (36)
Black or African American race	
No	1683 (64)
Yes	949 (36)
Race	
American Indian or Alaska Native	36 (1)
Asian	10 (< 1)
Black or African American	888 (34)
Native Hawaiian or Other Pacific Islander	8 (< 1)
White	1493 (57)
Multiracial	80 (3)
Unknown	117 (4)
Sexual Orientation	
Sexual minority (e.g., gay, lesbian, bisexual)	766 (29)
Straight or heterosexual	1866 (71)
Annual household income	
Less than $10,000	306 (12)
$10,000–$24,999	396 (15)
$25,000–$34,999	374 (14)
$35,000–$49,999	350 (13)
$50,000–$74,999	463 (18)
$75,000–$99,999	275 (10)
$100,000–$149,999	238 (9)
$150,000 or more	154 (6)
Prefer not to answer or unknown	76 (3)
Highest level of education	
Less than high school	92 (4)
High school diploma/equivalent	743 (28)
Some college	677 (26)
Associate degree or technical school	375 (14)
Bachelor’s degree/College graduate	470 (18)
Master’s degree	218 (8)
Doctoral degree	57 (2)
BMI category (kg/m^2^)	
Underweight (< 18.5)	178 (7)
“Normal” weight (18.5–24.9)	899 (34)
Overweight (25–29.9)	712 (27)
Obesity (≥ 30)	843 (32)

Participant race was assessed with the question: “How do you describe your race? Please check all that apply.” Response options included: American Indian or Alaska Native, Asian, Black or African American, Native Hawaiian or Other Pacific Islander, White, and Not listed (please specify). To create the variable Black or African American race, all participants who selected “Black or African American” for the question assessing race were coded as “yes,” including those participants who selected one or more additional response options, while participants who did not select “Black or African American” were coded as “no.” Percentages may exceed 100% due to rounding error

Descriptive statistics and correlations among perceived weight discrimination and outcome variables are presented in Table 2. The mean score on the SSI-B was 0.83 (SD = .83; range: 0–3), which corresponds to participants experiencing weight-related discrimination, on average, about once in their lifetime. Nevertheless, the relatively high standard deviation suggests there was substantial variability in participants’ degree of exposure. The most common cardiometabolic condition in the sample was hypertension, with one third of participants reporting a positive history. The second most common condition was high cholesterol, which was present in over 25% of the sample. Fourteen percent had diabetes. Fewer participants (7–12%) reported having a heart condition, stroke, or kidney disease. Except for kidney disease, the prevalence of each condition was significantly higher in men than women (data not shown). Scores on the cardiometabolic risk index (i.e., the number of cardiometabolic conditions reported by each participant) were as follows: over half the sample (51%) had 0 conditions, 23% had 1 condition, 13% had 2 conditions, 8% had 3 conditions, 3% had 4 conditions, 2% had 5 conditions, and less than one percent had all 6 conditions. As expected, small- to medium-sized positive correlations were observed across the six conditions. Perceived weight discrimination was significantly correlated with the cardiometabolic risk index (r = .32, *p* < 0.001) and all six conditions individually (range: r = .18 to r = .24; all *p*s < .001).

**Table 2:** Descriptive statistics and correlations among perceived weight discrimination, proposed mediators, and cardiometabolic outcomes

Variable	n	Range	*M*	*SD*	1	2	3	4	5	6	7	8	9	10	11
1. SSI-B	2620	0–3	0.83	0.83	–										
2. Smoking	2612	0–2	0.80	0.92	0.16*	–									
3. Alcohol use	2621	0–12	3.33	3.16	0.23*	0.25*	–								
4. Sleep disturbance	2631	1–5	2.91	1.06	0.33*	0.14*	0.08*	–							
5. High blood pressure	2518	0–1	0.34	0.47	0.18*	0.11*	0.09*	0.12*	–						
6. High cholesterol	2501	0–1	0.26	0.44	0.18*	0.07*	0.06*	0.07*	0.40*	–					
7. Heart condition	2517	0–1	0.12	0.33	0.24*	0.14*	0.13*	0.11*	0.26*	0.27*	–				
8. Stroke	2552	0–1	0.08	0.27	0.24*	0.08*	0.15*	0.08*	0.23*	0.21*	0.40*	–			
9. Kidney disease	2546	0–1	0.07	0.26	0.19*	0.08*	0.06*	0.07*	0.16*	0.22*	0.32*	0.33*	–		
10. Diabetes	2543	0–1	0.14	0.35	0.24*	0.11*	0.09*	0.07*	0.26*	0.36*	0.31*	0.29*	0.29*	–	
11. Cardiometabolic risk	2615	0–6	0.98	1.30	0.32*	0.15*	0.14*	0.13*	0.70*	0.71*	0.63*	0.57*	0.52*	0.65*	–

Weight discrimination was measured with the Stigmatizing Situations Survey-Brief (SSI-B). Smoking history was coded as 0 = *never smoker* (54%), 1 = *former smoker* (12%), or 2 = *current smoker* (34%). Alcohol use was measured with the Alcohol Use Disorders Identification Test (AUDIT-C). Sleep disturbance was measured with the 6-item PROMIS short form. Cardiometabolic risk was operationalized as the number of cardiometabolic conditions reported by each participant (i.e., high blood pressure, high cholesterol, a heart condition, kidney disease, stroke, diabetes)

*p < 0.01

Results from the adjusted linear regression analysis predicting cardiometabolic risk from perceived weight discrimination are presented in Table 3. More frequent exposure to weight discrimination was associated with higher cardiometabolic risk scores, adjusting for demographic characteristics and BMI. Cardiometabolic risk scores increased by .572 units for every one-unit change on the SSI-B. Similar to the pattern observed for the primary outcome, perceived weight discrimination was associated with increased odds of being diagnosed with each of the six cardiometabolic conditions. Odds ratios (OR) and 95% confidence intervals (CI) for perceived weight discrimination ranged from OR 1.90, 95% CI [1.69, 2.13] for high blood pressure to OR 2.76, 95% CI [2.32, 3.29] for stroke (all *p*s < .001). See supplemental materials for details.

**Table 3:** Results from the adjusted linear regression analysis predicting the cardiometabolic risk index from perceived weight discrimination

Variable	*b*	SE	95% CI for *b*		Beta	t	Partial *r*	*p*
			*LL*	*UL*				
SSI-B	0.572	0.029	0.515	0.629	0.365	19.702	0.365	< 0.001
Black	0.179	0.050	0.081	0.278	0.066	3.563	0.071	< 0.001
Latino	0.106	0.051	0.006	0.206	0.039	2.069	0.041	0.039
Gender (Woman)	− 0.251	0.049	− 0.347	− 0.156	− 0.096	− 5.147	− 0.102	< 0.001
Sexual minority	− 0.046	0.053	− 0.149	0.058	− 0.016	− 0.865	− 0.017	0.387
Age	0.031	0.002	0.027	0.035	0.297	15.473	0.295	< 0.001
Income	− 0.010	0.014	− 0.037	0.017	− 0.016	− 0.740	− 0.015	0.459
Education	0.029	0.019	− 0.008	0.066	0.033	1.534	0.031	0.125
BMI	0.000	0.003	-0.006	0.006	0.001	0.053	0.001	0.958

Perceived weight discrimination was assessed with the Stigmatizing Situations Survey-Brief (SSI-B). The model was adjusted for race, ethnicity, gender, sexual orientation, age, income, education, and BMI. *BMI* body mass index, *b* unstandardized regression coefficient, *SE* standard error, *CI* confidence interval, *LL* lower limit, *UL* upper limit, *Beta* standardized regression coefficient

**Fig. 1:**
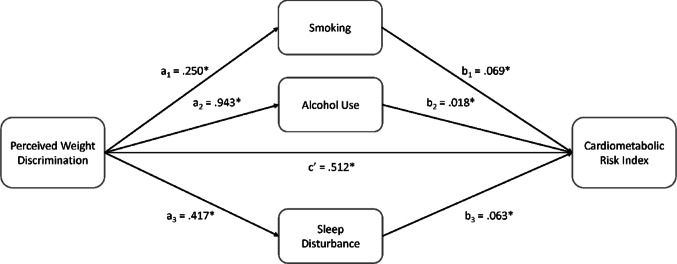
Results from the parallel multiple mediation model estimating the association between perceived weight discrimination and cardiometabolic risk as mediated by smoking, alcohol use, and sleep disturbance. Unstandardized regression coefficients are reported. None of the bootstrap confidence intervals for the indirect effects of smoking, a_1_b_1_ = 0.017, 95% CI [0.003, 0.032], alcohol use, a_2_b_2_ = 0.017, 95% CI [0.003, 0.033], or sleep disturbance, a_3_b_3_ = 0.026, 95% CI [0.010, 0.044] contained zero, providing evidence for mediation. * *p* < 0.05

Next, we tested whether smoking, alcohol use, and sleep disturbance mediated the association between perceived weight discrimination and cardiometabolic risk (Fig. 1). Results revealed that participants who were exposed to more frequent weight discrimination were significantly more likely to be current smokers, report more problematic alcohol use, and have higher sleep disturbance. In turn, more smoking, alcohol use, and sleep disturbance were all associated with significantly higher cardiometabolic risk scores. None of the confidence intervals for the indirect effects of smoking, a_1_b_1_ = .017, 95% CI [.003, .032], alcohol use, a_2_b_2_ = .017, 95% CI [.003, .033], or sleep disturbance, a_3_b_3_ = .026, 95% CI [.010, .044] contained zero, providing evidence that all three behavioral stress responses mediated the relationship between weight discrimination and cardiometabolic risk.

The final exploratory analysis assessed whether the relationship between perceived weight discrimination and cardiometabolic risk was moderated by race, ethnicity, gender, or sexual orientation. A statistically significant interaction was observed between SSI-B and gender, b = − .329, 95% CI [− .443, − .215], *p* < .001. Simple effects tests revealed that more frequent exposure to weight discrimination was associated with higher cardiometabolic risk in both men and women; however, the effect was significantly larger in men. The unstandardized regression coefficients and 95% CIs for the effect of perceived weight discrimination in men and women were b = .719, 95% CI [.642, .796], *p* < .001 and b = .390, 95% CI [.306, .474], *p* < .001, respectively. No additional interactions between demographic variables and perceived weight discrimination were observed.

## Discussion

Results indicated that more frequent exposure to weight-based discrimination was associated with worse cardiometabolic health. US adults who reported more frequent experiences with weight discrimination were more likely to be diagnosed with cardiometabolic conditions such as hypertension, stroke, or diabetes. Notably, the effect of perceived weight discrimination held while adjusting for BMI, suggesting that the health consequences of weight discrimination are independent of physiological factors associated with weight or body size. It is also important to acknowledge that these findings were observed in a sample of individuals from demographic groups that have historically been underrepresented in previous weight stigma research (i.e., participants who identify as Black or African American, Hispanic or Latino, or a sexual minority). These findings contribute to the growing literature on the negative health consequences of weight discrimination and suggest that the social experience of being stigmatized for one’s body weight or size may increase risk for cardiometabolic disease.

What might explain the association between perceived weight discrimination and cardiometabolic risk? Stress-related processes triggered by unfair or humiliating experiences involving weight are believed to underlie this association (Hatzenbuehler, 2009; Major et al., 2018; Pascoe & Richman, 2009; Pascoe et al., 2022; Tomiyama, 2014; Williams & Mohammed, 2009; Williams et al., 2019). Stigma-related stress can activate cognitive, behavioral, physiological, and biochemical changes that interact with one another to adversely affect health (Major et al., 2018; Tomiyama, 2019). Despite strong evidence linking smoking, alcohol use, and poor sleep behavior to cardiometabolic dysfunction (Chaput & Stranges, 2025; Direksunthorn, 2025; Gallucci et al., 2020; Messner & Bernhard, 2014; Meza et al., 2022; Piano et al., 2025), the current study is one of the first to investigate these behaviors as possible behavioral mechanisms underlying the link between perceived weight discrimination and poor cardiometabolic health. Results provided support for the proposed mediation model: individuals with greater exposure to weight discrimination were more likely to be current smokers, engage in more problematic drinking behavior, and report more disrupted sleep and those behaviors, in turn, predicted higher cardiometabolic risk. It is possible that people experiencing weight discrimination may turn to smoking, alcohol, or other substances as a coping strategy intended to relieve the psychological and physiological stress of being devalued for their weight or size. Although some research is consistent with this hypothesis (Gerend et al., 2023; Himmelstein et al., 2019; Klinck et al., 2020; Puhl et al., 2019; Sutin & Terracciano, 2017), more work is needed. Little is known about how weight discrimination may disrupt sleep. Stigma-related stress could impact sleep via cognitive mechanisms (e.g., rumination about negative weight-related experiences), psychological distress (e.g., depression, anxiety), or physiological processes (e.g., release of hormones; activation of the sympathetic nervous system) (Gerend et al., 2021; Han et al., 2012; Slopen et al., 2016). Another possibility is that internalized weight stigma, defined broadly as the extent to which individuals devalue themselves because of their body weight (Durso & Latner, 2008; Pearl & Puhl, 2018), could serve as a more proximate mediator between perceived weight discrimination and health risk behaviors (Pearl & Puhl, 2018; Puhl et al., 2020). Such pathways should be investigated in future research.

Exploratory analyses investigated whether the strength of the association between perceived weight discrimination and cardiometabolic health status varied by race, ethnicity, gender, or sexual orientation. The only statistically significant moderator identified was gender: Examination of the findings indicated that the effect of weight discrimination was present in both genders but larger in men than in women. It is unclear why the association between weight discrimination and cardiometabolic risk was stronger for men. A similar pattern was observed by Wellman and colleagues for the association between weight stigma consciousness (i.e., the extent to which individuals are aware that they may experience discrimination based on their weight or size) and binge eating (Wellman et al., 2019). Those researchers proposed that, relative to women, men may be less accustomed to experiencing discrimination based on weight (or gender) and thus may be less equipped to cope with the stress of such experiences (Wellman et al., 2019). It is notable that the few studies that have investigated demographic characteristics as moderators of the association between perceived weight discrimination and health outcomes (e.g., incident dementia, premature mortality) typically have not found evidence of moderation (Sutin et al., 2015, 2019). Findings from the present study are consistent with previous work. Although the negative cardiometabolic health effects of weight discrimination may be stronger in men than in women, they do not appear to be limited to any one particular demographic group. Results suggest that no demographic subgroup is immune to the cardiometabolic health consequences associated with experiencing weight discrimination. Additional research is needed to replicate these findings and identify what might explain the observed gender difference. Future work would also benefit from greater emphasis on understanding and addressing the health-related consequences of intersectional forms of stigma and discrimination, for example, experiencing discrimination because of one’s weight/size in conjunction with one’s race or ethnicity (Pearl et al., 2024).

The current findings have important implications for programs designed to reduce the harmful cardiometabolic effects of weight discrimination. Because efforts to change societal views on weight and body size have seen only limited success (Danielsdottir et al., 2010), it may be more fruitful to focus on specific settings. Health care, for example, is a common setting in which individuals report stigmatizing experiences due to their weight or size (Puhl, 2023; Tomiyama et al., 2018). Encouraging health care systems to adopt a weight-inclusive (vs. weight-centric) approach to care could help patients with high body weight feel more comfortable seeking health care and improve cardiometabolic health outcomes (Mauldin et al., 2022; Pearl et al., 2024). A weight-inclusive approach “rests on the assumption that everybody is capable of achieving health and well-being independent of weight, given access to non-stigmatizing health care. This approach challenges the belief that a particular BMI reflects a particular set of health practices, health status, or moral character” (Tylka et al., 2014). Weight-inclusive patient care requires a multi-level approach that addresses clinician behavior (e.g., reducing weight bias and negative assumptions about weight; encouraging clinicians to screen all patients for cardiometabolic health problems), the practice environment (e.g., creating a welcoming environment for people of all weights and sizes; making appropriately-sized medical equipment readily accessible), and the larger system (e.g., increasing health care access for all) (Mauldin et al., 2022; Tylka et al., 2014). Findings also underscore the importance of recognizing weight discrimination as an independent risk factor for cardiometabolic disease. As such, cardiometabolic disease prevention programs should address the harms of weight-based stigma and discrimination. One place to start is to ensure that public health messages promoting cardiometabolic health emphasize the importance of healthy behaviors and stress management practices for everyone rather than focusing solely on people with high body weight or large body size (Tomiyama et al., 2018). Overall, our findings suggest that reducing weight stigma through clinical and public health efforts may be an impactful strategy for improving cardiometabolic health outcomes.

It is critical to interpret the current findings in light of study limitations, which provide valuable directions for future research. Our primary outcome variable—the index of cardiometabolic risk—relied on self-reported diagnosis with six chronic conditions. Participants’ reports could have been subject to inaccurate recall or biased reporting. Further, given the survey-based nature of the study, we were not able to collect risk factors for cardiometabolic disease using more objective measures (e.g., glucose or lipid levels from a blood draw; actual measures of blood pressure or waist circumference). Future studies should aim to replicate these findings using medical history data obtained from medical records and objective measures of cardiometabolic disease risk. While the BRFSS smoking measure assessed respondents’ lifetime smoking history, our measure of alcohol use focused on the past year, and our measure of sleep disturbance was limited to the past seven days. As the cardiometabolic health effects associated with alcohol consumption and sleep disturbance accumulate over time, future studies should consider using measures that capture chronic alcohol- and sleep-related problems. Another limitation of this study was the cross-sectional design, which precludes our ability to ascertain the correct temporal order of the variables. Because all variables were assessed at a single time point, the observed mediation effects should be interpreted with caution, as they do not provide strong evidence of causal pathways. Most studies in this literature are cross-sectional, thus limiting our understanding of how the cardiometabolic health effects of weight discrimination unfold over time. Longitudinal studies are sorely needed. Finally, the study was limited to cisgender adults, thus findings may not generalize to individuals who identify as a gender minority. It will be important for future research to assess effects of weight discrimination on cardiometabolic health outcomes in transgender and non-binary individuals.

In closing, people subjected to weight-based discrimination are at increased risk for cardiometabolic disease. Importantly, the psychological and physiological stress associated with these stigmatizing experiences appears to contribute independent risk to cardiometabolic health over and above BMI and may be explained, in part, by behavioral stress responses to weight discrimination including smoking, alcohol use, and poor sleep. Further, although effects may be larger in men than women, results suggest that the negative cardiometabolic health consequences of weight discrimination are observed across individuals from different demographic groups. People with high body weight or large body size remain one of the most socially devalued groups in American society. Thus, the current findings have significant implications for the cardiometabolic health of millions of Americans.

## Data Availability

De-identified data, analytic code, and materials associated with the study are available in the Open Science Framework at: https://osf.io/5qsjc/?view_only=9bd700f8afea47cf8cf7ca1cc13574b1.
